# Gait variability predicts cognitive impairment in older adults with subclinical cerebral small vessel disease

**DOI:** 10.3389/fnagi.2022.1052451

**Published:** 2022-11-18

**Authors:** Peter Mukli, Sam Detwiler, Cameron D. Owens, Tamas Csipo, Agnes Lipecz, Camila Bonin Pinto, Stefano Tarantini, Adam Nyul-Toth, Priya Balasubramanian, Jordan R. Hoffmeister, Anna Csiszar, Zoltan Ungvari, Angelia C. Kirkpatrick, Calin I. Prodan, Andriy Yabluchanskiy

**Affiliations:** ^1^Oklahoma Center for Geroscience and Healthy Brain Aging, University of Oklahoma Health Sciences Center, Oklahoma City, OK, United States; ^2^Vascular Cognitive Impairment, Neurodegeneration and Healthy Brain Aging Program, Department of Neurosurgery, University of Oklahoma Health Sciences Center, Oklahoma City, OK, United States; ^3^International Training Program in Geroscience, Doctoral School of Basic and Translational Medicine/Department of Public Health, Translational Medicine and Physiology, Semmelweis University, Budapest, Hungary; ^4^Peggy and Charles Stephenson Cancer Center, University of Oklahoma Health Sciences Center, Oklahoma City, OK, United States; ^5^Department of Health Promotion Sciences, College of Public Health, University of Oklahoma Health Sciences Center, Oklahoma City, OK, United States; ^6^Department of Medicine, University of Oklahoma Health Sciences Center, Oklahoma City, OK, United States; ^7^Veterans Affairs Medical Center, Oklahoma City, OK, United States; ^8^Department of Neurology, University of Oklahoma Health Sciences Center, Oklahoma City, OK, United States

**Keywords:** cerebral small vessel disease, gait variability, white matter hyperintensities, gait, cognitive dysfunction

## Abstract

**Introduction:**

Advanced methods of gait research, including approaches to quantify variability, and orderliness/regularity/predictability, are increasingly used to identify patients at risk for the development of cognitive impairment. Cerebral small vessel disease (CSVD) is highly prevalent in older adults and is known to contribute to the development of vascular cognitive impairment and dementia (VCID). Studies in preclinical models demonstrate that subclinical alterations precede CSVD-related cognitive impairment in gait coordination. In humans, CSVD also associates with gait abnormalities. The present study was designed to test the hypothesis that increased gait variability and gait asymmetry predict a decline in cognitive performance in older adults with CSVD.

**Methods:**

To test this hypothesis, we compared cognitive performance and gait function in patients with CSVD (age: 69.8 ± 5.3 years; *n* = 11) and age- and sex-matched control participants (age: 70.7 ± 5.8 years; *n* = 11). Based on imaging findings, patients with CSVD were identified [presence of white matter hyperintensities plus silent brain infarcts and/or microhemorrhages on magnetic resonance imaging (MRI) assessment]. Cognitive performance was assessed using the Cambridge Neuropsychological Test Automated Battery (CANTAB). Gait parameters were measured during the single and dual tasks, during which participants, in addition to the motor task, completed a series of mental arithmetic calculations. Spatial and temporal parameters of gait variability, symmetry, and permutation entropy were determined using a pressure-sensitive gait mat during single and dual cognitive task conditions.

**Results:**

Patients with CSVD exhibited lower performance in a visual learning test (*p* = 0.030) and in a sustained attention test (*p* = 0.007). CSVD also affected step time variability (*p* = 0.009) and step length variability (*p* = 0.017). Step lengths of CSVD participants were more asymmetric (*p* = 0.043) than that of controls, while the two groups were statistically similar regarding step time symmetry and entropy of step time and length. Gait variability was inversely associated with sustained attention, especially among CSVD patients, and this relationship was significantly different between the two groups. The association of sustained attention with gait symmetry was also significantly different between the two groups.

**Discussion:**

Our findings provide additional evidence in support of the concept that increased gait variability and asymmetry may predict cognitive impairment in older adults with CSVD.

## Introduction

Cerebral small vessel disease (CSVD) is an age-related pathological condition affecting cerebral microvessels, which manifests as white matter hyperintensities (WMHs), small subcortical infarcts and lacunes and/or cerebral microhemorrhages on brain imaging. By causing both white matter injury and damage to the neurons located in the cortex, hippocampi and cerebral nuclei CSVD promotes the development of vascular cognitive impairment and dementia (VCID) (Debette and Markus, 2010; Prins and Scheltens, 2015), which is the second most common type of dementia after Alzheimer’s disease (Wardlaw et al., 2015; Corriveau et al., 2016; Gorelick et al., 2016). There is an urgent need to develop novel screening methods to identify patients at risk for CSVD-induced cognitive decline.

Regulation of human gait involves multiple brain regions and a complex interaction of motor and cognitive processing. Impairments in multiple cognitive domains in older adults due to various underlying pathologies (ranging from the effects of aging *per se* to Alzheimer’s disease) were shown to strongly associate with complex alterations in gait (Goldberger et al., 2002; Owings and Grabiner, 2004; Hausdorff, 2005; Hollman et al., 2007; Brach et al., 2008, 2010; Montero-Odasso et al., 2014; Rosso et al., 2015; Sorond et al., 2015; Cohen et al., 2016; Moon et al., 2016; Hackett et al., 2018; Öhlin et al., 2020) shown to associate with multifaceted gait abnormalities, including slower speed and balance function (Bäzner et al., 2000; Baezner et al., 2008; de Laat et al., 2010; Choi et al., 2012; Smith et al., 2015; Kim et al., 2016; Pinter et al., 2017a,b; Su et al., 2022). In the past decade, advanced methods of gait research, including approaches to quantify variability and regularity/predictability/probability have been introduced to identify patients at risk for the development of cognitive impairment (Savica et al., 2017; Windham et al., 2022). Studies in preclinical models confirm that CSVD-related cognitive impairment is preceded by subclinical alterations in gait coordination, including increased gait variability, decreased symmetry and decreased orderliness and regularity (Toth et al., 2015; Tarantini et al., 2017; Nyul-Toth et al., 2020, 2021, 2022). Despite these advances, the effects of CSVD on gait variability, symmetry and orderliness in older adults are not well-characterized.

The present study was designed to test the hypotheses that older adults with CSVD exhibit increased gait variability, decreased symmetry and/or decreased orderliness and that these subclinical gait alterations predict cognitive decline. To test these hypotheses, we enrolled older adults with imaging signs of CSVD and compared their performance on cognitive tests, gait variability, symmetry, and orderliness during single and dual task to those of age- and sex-matched controls. The associations between these gait parameters and cognitive performance and the impact of CSVD on these relationships were also analyzed.

## Materials and methods

### Study design and participant characteristics

A total of 28 English-speaking adults [14 participants with CSVD, 71.6 ± 6.9 years of age, 11 males (all White) and three females (one African American and two White); and 14 controls, 71.7 ± 6.5 years of age, 11 males (all White) and three females (one African American and two White);*p* = 0.97, Mean ± SD] were enrolled in this cross-sectional study. Participants were recruited through the VA Oklahoma City Health Care, the Department of Neurology, or the Translational Geroscience Laboratory at the University of Oklahoma Health Sciences Center. The CSVD patient group was defined by the presence of silent brain infarct(s) and/or cerebral microhemorrhages (defined as bleed size ≤5 mm), the presence of periventricular or deep WMH revealed by magnetic resonance imaging (MRI), and a diagnosis of mild cognitive impairment based on Montreal Cognitive Assessment (MoCa, inclusion range: ≥22) (Nasreddine et al., 2005). To confirm the presence of WMH, a neurological expert evaluated the MRI and determined if the sum of the Fazekas-scale for the periventricular and deep white matter is greater or equal to two. To date, it is the most widely used system for describing white matter disease severity in the field (Fazekas et al., 1987; Andere et al., 2022). We also assessed the number of cerebral microbleeds and silent brain infarcts in the cortical and subcortical brain structures separately. Participants with a medical history of major cerebrovascular events were excluded from the final sample selection and were well-controlled for any comorbidities that could have affected the endpoints. In addition, participants were screened for orthopedic complications and were excluded if orthopedic conditions were a limiting factor in their walking ability.

All of the 14 patients with CSVD and all of 14 older adults in the control group completed single and dual task gait tests, and 12 from each group were administered a battery of neuropsychological tests. All participants were asked to refrain from drinking caffeinated beverages for at least 6 h before the assessments. All participants were right-handed. All procedures and protocols were approved by the Institutional Review Board of the University of Oklahoma Health Sciences Center. Informed consent was obtained from each participant prior to participation.

### Cognitive assessment using standardized battery of neuropsychological tests

Attention, psychomotor speed, executive function, and immediate memory were evaluated using a selection of tests from the Cambridge Neuropsychological Test Automated Battery (CANTAB, Cambridge Cognition). The CANTAB Connect Research tool was used and is a validated and sensitive tool optimized to detect signs of mild cognitive decline and age-related cognitive impairment (Abbott et al., 2019; Csipo et al., 2019). Testing began with a Motor Screening Task to determine if any sensorimotor deficits were present and proceeded with the following tests: Reaction Time, Paired Associates Learning, Delayed Matching Sample, Spatial Working Memory, Rapid Visual Processing and Attention. For these tests, participants were left alone in a quiet space with a touchscreen device running the application.

### Gait assessment

Gait function was assessed using the Zeno Walkway System (ProtoKinetics, Havertown, PA, USA), which provides a standardized comprehensive characterization of gait variability (Vallabhajosula et al., 2019). Testing began with the participant being asked to walk back and forth across the length of the mat, completing ∼70–80 steps. During the single task, participants were instructed to walk with their arms out of their pockets and swinging normally.

Next, participants were instructed to take the same amount of walking sessions during a dual task. Due to its sensitivity to cognitive workload-related change in gait variability, a mental arithmetic paradigm (Beauchet et al., 2005; Allali et al., 2007; Radovanović et al., 2016) was administered. In brief, participants were asked to subtract seven consecutively from 500 (examples of correct responses: 500, 493, 486, 479.).

Based on the location of pressure changes, we collected data on distance, the duration between two successive steps (step length and step time, respectively), and for each walking cycle (stride length and stride time, respectively), yielding time series for each gait parameter. While the utilized electrical mat can comprehensively assess gait patterns, we chose parameters—described in the subsequent section in more detail—reflecting gait variability, symmetry, and entropy in line with our hypotheses.

### Analysis of gait pattern

For gait variability, mean, standard deviation and coefficient of variation (CV, defined as the ratio of standard deviation and mean) were calculated for the time series of these spatial or temporal parameters of the gait pattern. For further analysis, we considered CV as the most unbiased measure of gait variability since it takes into account differences related to the body size of the study participant in the corresponding standard deviation of the spatial and temporal parameters. Step time and step length CV were calculated for left, right and both feet.

Our previous investigations revealed gait asymmetry in animal models of cerebral microhemorrhages (Nyul-Toth et al., 2020) with the aid of the symmetry index (*SI*). Therefore, we used SI for the assessment of gait symmetry that is derived from series of spatial or temporal gait characteristics as follows (Tarantini et al., 2019):


(1) $$SI=\frac{2\cdot \left|X_{left}-X_{right}\right|}{\left(X_{left}-X_{right}\right)}$$

where *X*_*left*_ and *X*_*right*_ are the mean of step time or step length values, respectively.

The orderliness of physiological processes can be characterized model-freely using entropy measures (44), which could also be applied to gait dynamics. Several methods have been proposed to estimate entropy based on gait sequence data (Deffeyes et al., 2009; McCamley et al., 2018), such as sample entropy and approximate entropy, which are rather sensitive to signal length (Yentes et al., 2013; Yentes and Raffalt, 2021). Based on the concept of these methods, Bandt and Pompe (2002) introduced permutation entropy (PE), which was shown to be applicable for short data sets (Riedl et al., 2013) and has the advantage that it requires fewer input parameters compared to the aforementioned methods, namely the embedding dimension (*m*) and time lag (*L*).

Permutation entropy calculation begins with embedding a discretely sampled time series *x*(*t*) = *x*_1_,*x*_2_,…,*x*_*N*_ of length *N*—in an *m*-dimensional state space as a sequence of state space vectors *X*(*t*) using a temporal embedding scheme (Takens, 1981). Specifically, when using an embedding dimension and embedding time lag *L*, *X*(*t*) is defined as


(2) $$X(t)=\left[x_{t},x_{t-L},\ldots ,x_{t-(m-1)L}\right]$$

Then values in each embedding vector *X*(*t*) are converted to ranks, converting *X*(*t*) (which has length *m*) into one of the *m*! possible permutations of rank *m*. The relative frequency of each permutation/order sequence is calculated as


(3) p(π)=# of⁢ type π permutationT−(m−1)L+1

yielding an estimate of the probability distribution of each permutation type in the process (Bandt and Pompe, 2002; Racz et al., 2019). Finally, permutation entropy of order *m*, *H*(*m*) is defined (Bandt and Pompe, 2002) as


(4) $$H(m)=-\sum_{\pi \in \prod }p(\pi )\log p(\pi )$$

where Π denotes the set of *m*! possible permutations of rank *m*. From equation (3) and the concept of information theoretical entropy, it follows that *H*(*m*) has a theoretical maximum of *log*⁡*m*!, marking the scenario where all permutations occur with equal probability, thus resulting in maximal entropy (Bandt and Pompe, 2002). Therefore, PE can be “normalized” to be in the range [0,1], where 0 and 1 indicate complete predictability (minimal complexity) and maximal unpredictability (maximal complexity), respectively. In most studies, the input parameters *m* and *L* of PE are set according to empirical or practical considerations rather than being defined by the characteristics of the data itself (Staniek and Lehnertz, 2007; Li et al., 2013). In case of gait, the typical value of *m* is three and *L* is one (Stergiou et al., 2004; Riedl et al., 2013).

### Statistical analysis

The key dependent variables of the present clinical study were neuropsychological parameters yielded by CANTAB and CV, SI and PE calculated for Step Time (subscript _T_) and Step Length (subscript _L_). To test whether samples of these cognitive and gait parameters in the Control (CONT) and CSVD groups follow Gaussian distribution, we used Shapiro-Wilk’s method with a significance level of *p* < 0.05. In the case of normal distributions, we used Levene’s test to assess the homogeneity of variances with a significance level of *p* < 0.05.

If samples followed Gaussian distribution and their variances were homogenous, we used two-way repeated measures ANOVA (RM-ANOVA) with CSVD/CONT group and single/dual task condition as a categorical factor and repeated measures, respectively. Subsequently, significant differences were checked by Šídák’s multiple comparisons test. In cases of heterogeneous variance between groups and non-normal distributions that were resistant to logarithmic transformations, we used the Mann-Whitney U test to compare the CONT and CSVD groups and Wilcoxon-test to compare gait parameters obtained during single and dual task conditions.

To test whether parameters of gait pattern associate with cognitive outcome measures, we fitted a general linear model (GLM) to the data for normal distributions and homogeneous variances. We used separate slopes model, which incorporated CONT/CSVD groups as a categorical factor and *CV*_T_ or *CV*_L_ as a continuous predictor and their interaction (gait parameter x group) as well. This assessed the impact and significance of group-independent and specific association between gait parameters and cognitive performance. Finally, we performed a correlation analysis for the CONT, CSVD and study population yielding Pearson’s (in case of normal distribution) or Spearman’s correlation (non-normal distributions) coefficient (*r*) and significance. This shows how much variance of the examined gait parameter explains variance in the examined cognitive outcome measure and whether there is a significant relationship between these variables.

## Results

### Patient characteristics and neuroimaging data

Details of the study population can be found in Table 1, while the MRI findings are summarized in Table 2.

**TABLE 1:** Characteristics of the study population.

Health conditions	CSVD (*n* = 11, 59–78 years old, three females, eight males)	Control (*n* = 11, 65–83 years old, two females, nine males)
Depression	4/11	2/11
T2D	5/11	1/11
Hypertension	9/11	6/11
Hyperlipidemia	8/11	6/11
CAD	5/11	0/11
Other conditions	arthritis (1), gout (1), PTSD (2), DJD (2), PAD (2), carotid artery stenosis (3), atrial fibrillation (3)	arthritis (2), DJD (1), osteoporosis (2), asthma (1), peripheral neuropathy (1), type 1 diabetes (1)

Comorbidities considered as potential confounders are reported for cerebral small vessel disease (CSVD) patients and control group. All participants with comorbidities were medically controlled for by their physician: allopurinol, aspirin, bupropion, zolpidem, atorvastatin, apixaban, metoprolol, citalopram, lisinopril, sildenafil, amlodipine, metformin, rivaroxaban, hydrochlorothiazide, valsartan, albuterol, alendronate, eszopiclone, insulin detemir, insulin aspart. PTSD, Post-traumatic stress disorder; DJD, Degenerative joint Disease; T2D, Type 2 diabetes; PAD, Peripheral artery disease; CAD, Coronary artery disease.

**TABLE 2:** Summary of magnetic resonance imaging (MRI) findings.

MRI pathology present (left, right)	CSVD (*n* = 11)
Cortical SBI	0/11 (0, 0)
Subcortical SBI	8/11 (8, 5)
Lobar microbleeds	2/11 (2, 0)
Deep microbleeds	6/11 (2, 2)
Mixed microbleeds	1/11 (1, 1)
WMH fazekas scale ≥ 2	11/11

MRI, magnetic resonance imaging; SBI, silent brain infarct; WMH, white matter hyperintensity.

Three participants were excluded from the CSVD group due to the lack of silent brain infarct or cerebral microhemorrhages (defined as bleed size ≤5 mm), together with three CONT subjects to maintain matching in terms of sample size, mean age and distribution of sex. The number of silent brain infarcts ranged from 0 to 5, all in subcortical distribution, and more numerous in the left than the right hemisphere (mean number of silent brain infarcts 1.2 versus 0.6). The number of microbleeds ranged from 0 to 2, most often deep (Table 2), and also slightly favoring the left versus right hemisphere (total six versus four microbleeds). WMH was found to be distributed evenly among both hemispheres.

### Effects of cerebral small vessel disease on cognitive performance in older adults

The key cognitive outcome measures for each cognitive domain are reported in Table 3.

**TABLE 3:** Comparison of Cambridge Neuropsychological Test Automated Battery (CANTAB) between cerebral small vessel disease (CSVD) patients and age-matched controls (CONT).

		Statistic	*df*	*P*	Effect size
**PALFAMS28**	Student’s-*t*	1.994	20	**0.03**	0.8501
RTIFMDMT	Student’s-*t*	–1.151	17	0.867	–0.5286
RTIFMDRT	Student’s-*t*	–2.058	17	0.972	–0.9458
**RVPA**	Student’s-*t*	2.806	17	**0.007**	1.2892
RVPMDL	Mann-Whitney U	36		0.777	0.2
*SWMBE468*	Student’s-*t*	–0.483	20	0.683	–0.2059
SWMS	Mann-Whitney U	56		0.393	0.0744
Counting: Correct#	Student’s-*t*	1.163	20	0.13	0.52

Bold denotes significant differences (*p* < 0.05, one-sided test: error scores—shown in italic—were expected to be higher in the CSVD group, performance scores were expected to be higher in the CONT group). PALFAMS28, paired association learning first attempt memory score; RTIFMDMT, RTI simple median movement time; RTIFMDRT, RTI simple median reaction time; RVPA, rapid visual processing and attention score; RVPMDL, rapid visual processing median response latency; SWMS, spatial working memory strategy score; SWMBE468, spatial working memory within errors; Correct#, measure of mental arithmetic performance during dual task. Unpaired comparisons revealed significant differences for PALFAMS28 and RVPA score.

**FIGURE 1:**
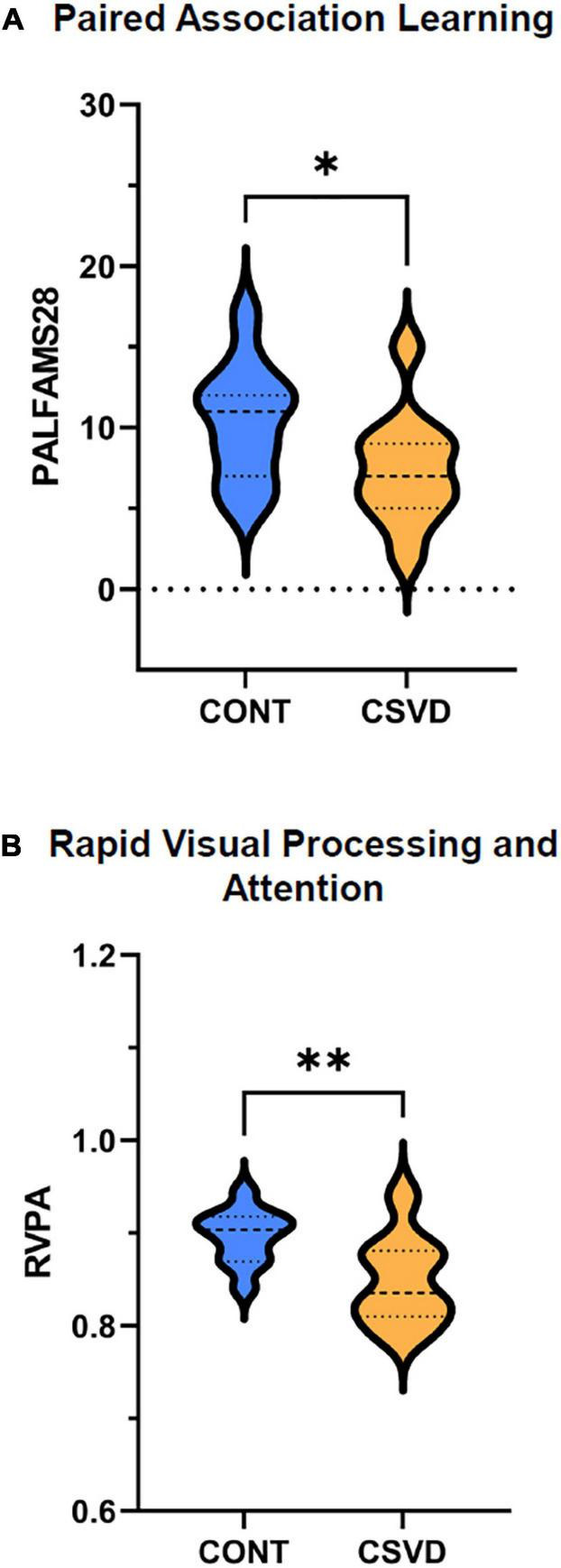
Cognitive performance is impaired in older adults with cerebral small vessel disease (CSVD). **(A)** We observed a significant decrease in the Paired Associate Learning First Attempt Memory Score (PALFAMS28) in the patients versus age- and sex-matched elderly controls (CONT), evidenced as a significantly higher percentage of recalling the correct box at first attempt (two-sample unpaired *t*-test, *p* = 0.03, *n* = 11 per group). **(B)** We also observed a significantly decreased performance in the rapid visual processing and attention (RVPA) test (0 to 1 from poor to good performance; two-sample unpaired *t*-test, *p* = 0.007, CONT: *n* = 11, CSVD: *n* = 9) in participants with CSVD compared with CONT. Dashed line mark the median and dotted lines bound the interquartile range. *p < 0.05, **p < 0.01.

We observed statistically significant group differences in the performance of a Paired Associates Learning test (Figure 1A, 10.360 ± 3.585 vs. 7.364 ± 3.472; mean ± SD of first attempt memory score, *p* = 0.030), indicating that CSVD patients demonstrated poorer ability to encode and retrieve newly formed visual associations. We also observed a significantly worse performance on a Rapid Visual Processing and Attention (RVPA) test in CSVD participants (Figure 1B, 0.8968 ± 0.0340 vs. 0.8487 ± 0.0483; mean ± SD, *p* = 0.007), indicating that CSVD in older adults is associated with poorer sustained attention.

### Cerebral small vessel disease increases step time and step length variability in older adults

**FIGURE 2:**
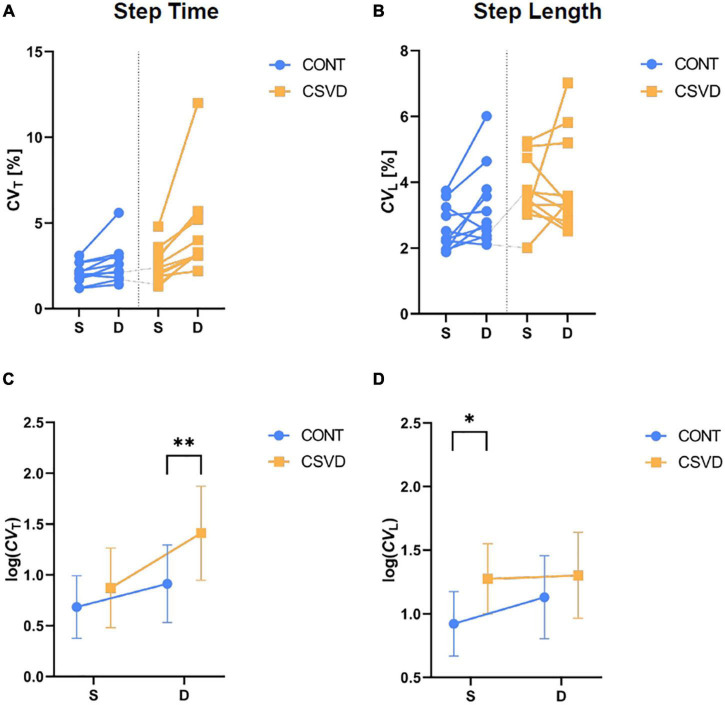
Alterations in gait variability among older adults with cerebral small vessel diseases (CSVD) during single (S) and dual (D) task conditions. The impact of task condition on Coefficient of Variation (CV) of Step Time [*CV*_T_, panel **(A)**] and Step Length [*CV*_L_, panel **(B)**] are shown for CSVD (*n* = 11; orange square symbols) and age- and sex-matched control (CONT, *n* = 11; light blue circle symbols) groups, respectively. **(C)** Log-transformed *CV*_T_ significantly differs between CONT and CSVD group for dual task condition (two-way RM-ANOVA, *p* = 0.006; Šidák’s multiple comparisons test, *p* = 0.009). **(D)** Log-transformed *CV*_L_ significantly differs between CONT and CSVD group (two-way RM-ANOVA, *p* = 0.006) for single task conditions (Šidák’s multiple comparisons test, *p* = 0.017). Symbol and error bar depict mean ± SD. *p < 0.05, **p < 0.01.

We calculated the *CV* of Step Time (*CV*_T_) and Step Length (*CV*_L_) from gait recorded during single and dual task conditions for both feet. For statistical analysis and displaying purposes, we took the natural logarithm of *CV*_T_ and *CV*_L_ as dependent variables. It allowed us to use two-way repeated measures ANOVA with group as an independent categorical factor, while task condition was a repeated measures factor in the model with two levels (single and dual task). Figure 2A shows that the dual task induces a larger change in *CV*_T_ (CONT vs. CSVD: 3.259 ± 1.187 vs. 3.887 ± 1.463; mean ± SD of *CV*_T_ % values) among CSVD patients yielding a better separation of the two groups (two-way RM-ANOVA for log(*CV*_T_), the effect of task: *p* = 0.002). Accordingly, the group effect was significant only for dual task condition (Figure 2C). For *CV*_L_ (Figures 2B,D), we found a significant difference between CONT and CSVD (two-way RM-ANOVA, the effect of group: *p* = 0.006; Šidák’s multiple comparisons test, *p* = 0.009) only in the single task condition (CONT vs. CSVD: 2.591 ± 0.681 vs. 3.699 ± 0.9744; mean ± SD of CV % values). Dual task condition did not separate the two groups but had a significant effect on *CV*_L_ (two-way ANOVA, the effect of task: *p* = 0.013; Šidák’s multiple comparisons test, *p* = 0.017).

From the same recordings, we analyzed stride times and stride lengths which measure the distance and duration of one stride (data not shown). We found that only the dual task had a statistically remarkable effect on these gait variability parameters. Finally, we determined unilateral gait variability parameters, which were not influenced significantly by CSVD except for right foot *CV*_L_.

### Effects of cerebral small vessel diseases on gait symmetry in older adults

**FIGURE 3:**
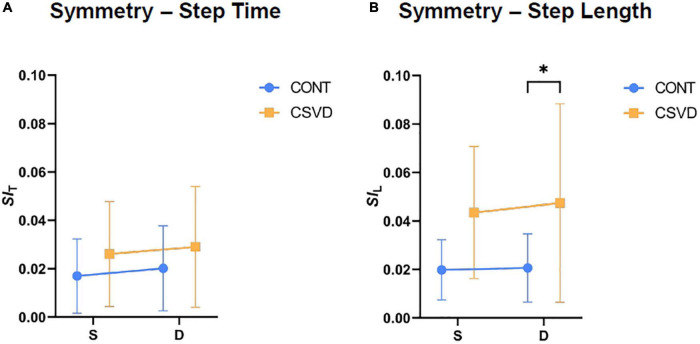
Gait symmetry is altered by cerebral small vessel disease (CSVD). **(A)** Symmetry Index of Step Time (*SI*_T_) is statistically similar (Mann-Whitney test, *p* > 0.05) between CSVD group (*n* = 11; orange square symbols) and age- and sex matched control (CONT, *n* = 11; light blue circle symbols) for both task conditions. **(B)** Symmetry Index of Step Length (*SI*_L_) significantly differs between CONT and CSVD group but only for dual task condition (Mann-Whitney test, *p* > 0.019). Symbol and error bar depicts mean ± SD. *p < 0.05.

We calculated indices of gait symmetry from Step Length and Step Time recordings based on Eq. 1. Mann-Whitney U tests did not reveal a significant difference in the Step Time symmetry index (*SI*_T_) of the CSVD group compared to age- and sex-matched controls (Figure 3A) neither during single (0.021 ± 0.015 vs. 0.024 ± 0.018; mean ± SD) nor during dual task conditions (0.021 ± 0.020 vs. 0.025 ± 0.024; mean ± SD). In contrast, Step Length asymmetry (denoted as *SI*_L_) was higher in elderly patients with CSVD (Figure 3B) compared to controls during single (0.020 ± 0.013 vs. 0.041 ± 0.025; mean ± SD) and during dual task conditions (0.023 ± 0.015 vs. 0.047 ± 0.038; mean ± SD) showing a significant difference between the two groups. The increase in symmetry indices associated with dual task was non-significant in cases of Step Time and Step Length (Wilcoxon-test, *p* > 0.05), respectively.

### Effects of cerebral small vessel diseases on gait entropy in older adults

**FIGURE 4:**
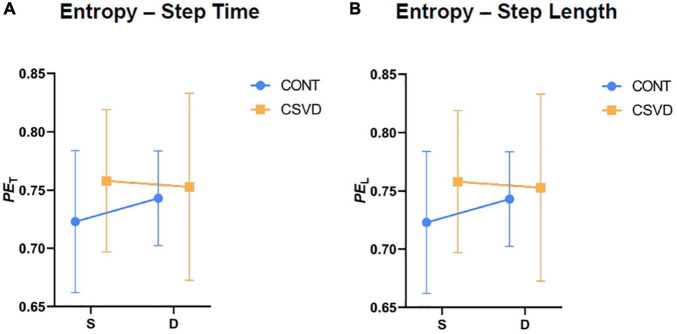
Entropy of gait pattern is not altered by cerebral small vessel disease (CSVD). Permutation Entropy of Step Time [*PE*_T_, panel **(A)**] and Permutation Entropy of Step Length [*PE*_L_, panel **(B)**] are statistically similar (two-way RM-ANOVA, *p* > 0.05) between CSVD (*n* = 11; orange square symbols) and age- and sex matched elderly control (CONT, *n* = 11; light blue circle symbols) groups for both task conditions. Symbol and error bar depicts mean ± SD.

We calculated PE of gait from Step Length (denoted as *PE*_T_) and Step Time (denoted as *PE*_L_) recordings based on Eq. 4. Figure 4 shows that *PE*_T_ (panel A) and *PE*_L_ (panel B) increase during dual task for control participants. In contrast, elderly patients with CSVD have a higher entropy compared to controls during single task and dual-task shifts their gait entropy towards a pattern with lower permutation entropy. However, two-way RM-ANOVA revealed that these changes are not statistically significant (*p* > 0.05).

### Higher gait variability associates with impaired cognitive performance in older adults with cerebral small vessel diseases

**FIGURE 5:**
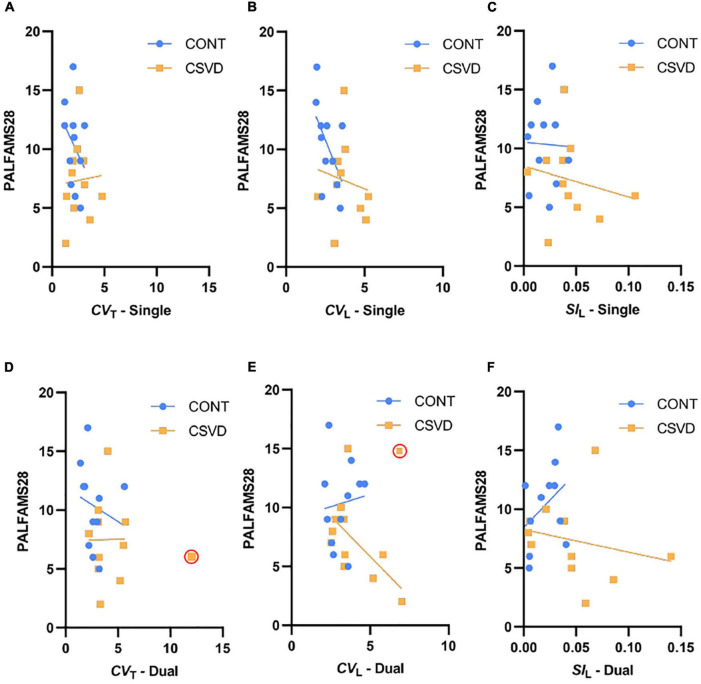
Relationship between gait parameters and Paired Association Learning First Attempt Memory (PALFAMS28) score. PALFAMS28 scores are plotted as a function of Step Time Coefficient of Variation [*CV*_T_, panels **(A,D)**], Step Length Coefficient of Variation [*CV*_L_, panels **(B,E)**] and Step Length Symmetry [*SI*_L_, panels **(C,F)**]. Data of patients with cerebral small vessel disease (CSVD, *n* = 11) are shown as orange squares while those of age- and sex-matched control participants (CONT, *n* = 11) are shown as light blue circles. Top and bottom panels display gait parameters during single and dual task conditions respectively. Trendlines are displayed for each group separately with the corresponding color. PALFAMS28 scores were not associated with any of the gait parameters.

**FIGURE 6:**
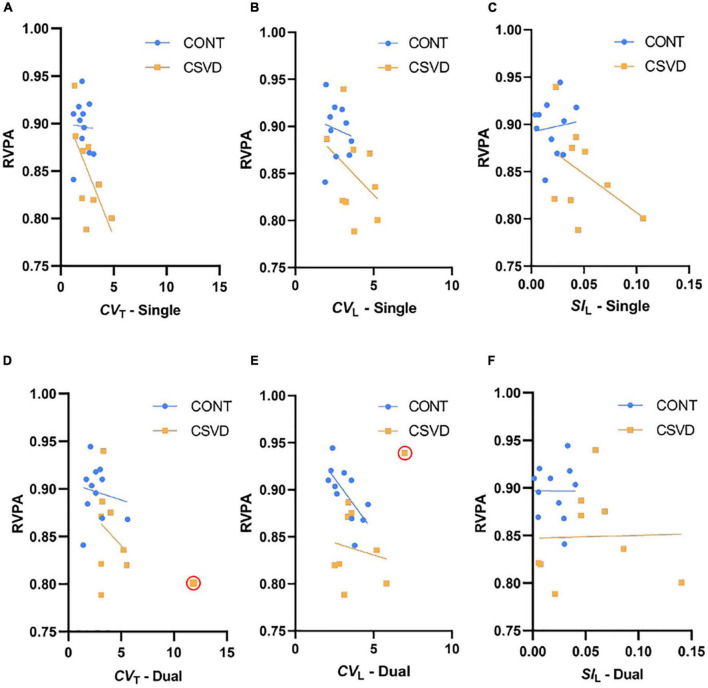
Relationship between gait parameters and Rapid Visual Processing and Attention (RVPA) score. RVPA scores are plotted as a function of Step Time Coefficient of Variation [*CV*_T_, panels **(A,D)**], Step Length Coefficient of Variation [*CV*_L_, panels **(B,E)**] and Step Length Symmetry [*SI*_L_, panels **(C,F)**]. Data of patients with cerebral small vessel disease (CSVD, *n* = 9) are shown as orange squares while those of age- and sex-matched control participants (CONT, *n* = 11) are shown as light blue circles. Top and bottom panels display gait parameters during single and dual task conditions, respectively, outlier values are denoted by red circle. Trendlines are displayed for each group separately with the corresponding color. Please note that CSVD patients with higher Step Time Coefficient of Variation (*CV*_T_) during single task condition had a lower RVPA score compared to age- and sex-matched controls: Pearson’s *r* = –0.652 and *p* = 0.057 in CSVD group compared to Pearson’s *r* = –0.030 and *p* = 0.909 in CONT group. Such pattern is also visible for the other gait parameters particularly during single task condition but not in dual task condition, however the impact of the continuous predictor and CSVD were neither significant in any of these relationships.

To investigate the relationship between cognition and gait in our study population, we correlated measures of cognitive performance with gait variability and symmetry, which differed between CSVD and CONT groups. No significant relationship was found between the Paired Associates Learning test, a measure of visual learning abilities, and step time/length variability or asymmetry (Figure 5). In contrast, lower RVPA scores were associated with higher *CV*_T_ (Figure 6A) during the single-task condition [GLM: *p* = 0.040, the effect size of continuous predictor (η*): 0.224] which was also reflected by their significant correlation (Pearson’s *r* = –0.544, *p* = 0.013). In particular, the effect of CSVD was significant on the *CV*_T_ -RVPA relationship [GLM: *p* = 0.040, the effect size of the group (η*): 0.225]. Rank correlation analyses (justified by the heterogeneous variance) did not reveal a significant relationship between RVPA score and *CV*_L_ (Figure 6B), neither in CSVD nor in CONT groups. However, this relationship is present in the whole sample (Pearson’s *r* = –0.530, *p* = 0.016). Similarly, the gait variability parameters corresponding to dual task conditions (Figures 6D,E) did not relate to RVPA score in the CSVD group but only in the CONT group for *CV*_L_ (Spearman’s *r* = –0.756, *p* = 0.009). Patients with more asymmetric gait during single task condition (Figure 6C) likely performed more poorly in the RVPA test, but *SI*_L_ or CSVD did not have a significant impact on RVPA score, which was indeed clearly absent for dual task condition (Figure 6F).

## Discussion

In this cross-sectional study, we tested the hypothesis that CSVD associated with poor cognitive performance and gait abnormalities, focusing on tests and parameters sensitive to subtle changes in these functional domains. To evaluate the predictive value of gait parameters, the impact of CSVD on gait variability, entropy and symmetry during mental workload was studied, and the results were correlated with cognitive performance.

We found that patients with CSVD exhibited impaired cognitive performance, extending the findings of previous studies (Zanon Zotin et al., 2021). A detailed cognitive evaluation of our study participants revealed that the presence of subclinical cerebrovascular lesions—albeit heterogeneously localized—was associated with significant deficits in their attention and immediate memory performance (Figure 1). Such cognitive deficits are characteristic of the clinical picture of CSVD (Prins et al., 2005; van Norden et al., 2011; Kynast et al., 2018). Although WMH is often found in aged persons without cognitive symptoms (Longstreth et al., 1996; De Leeuw et al., 2001; Elahi et al., 2018), it is more common in patients with dementia or predementia states (Prins and Scheltens, 2015; Ungvari et al., 2017b; Szczesniak et al., 2021). Both cerebral microhemorrhages have been shown to promote VCID and hold a significant role in pathological brain aging (Ungvari et al., 2017a; Azeem et al., 2020). The finding that CSVD associated with attention deficits is of particular interest since it indicates impairment of cognitive capacities that are necessary for gait control.

Our study provides new evidence that older adults with CSVD exhibit alterations in gait patterns (Hairu et al., 2021; Ma et al., 2022) characterized primarily by increased variability and asymmetry. Locomotion represents the integrated output of multiple regulatory centers in the central nervous system. The impaired structural integrity of any of these brain regions may alter physiological gait patterns (Fitzpatrick et al., 2007; Zhang et al., 2019), especially in aged persons with a higher degree of cognitive involvement in gait control (Li et al., 2018). Gait variability is a particularly sensitive parameter, affected by chronological aging itself (Hollman et al., 2007; Brach et al., 2008, 2010; Callisaya et al., 2010). The age-related changes in *CV*_T_ can be exacerbated in pathological conditions associated with damage to the brain gait control network, such as CSVD. The finding that *CV*_T_ was significantly increased in older adults with CSVD during dual task condition is consistent with the concept that an additional cognitive task administered during gait tests (Beauchet et al., 2009; Ghanavati et al., 2018) increases the sensitivity of the method to detect CSVD-related subclinical brain dysfunction (Beauchet et al., 2005; Allali et al., 2007; Hollman et al., 2007). Attention is particularly implicated in gait control and simultaneous mental activities engaging this cognitive domain compete with each other, resulting in cognitive-motor interference and altered gait patterns (Koren et al., 2022). In the present study, we also assessed gait uniformity by determining a sensitive, spatial measure of gait symmetry (*SI*_L_) (Tarantini et al., 2019) which has been validated in preclinical models of CSVD (Nyul-Toth et al., 2020). The findings that older adults with CSVD exhibit subtle gait asymmetry likely correspond to the uneven bilateral distributions of WMHs, CMHs, and lacunar infarcts in the two hemispheres as seen on MRI images. Indeed, statistical analysis revealed significant correlation between the laterality of these MRI pathologies and step time asymmetry during dual task (*p* = 0.012). Our results extend the findings of recent studies demonstrating a significantly asymmetric pattern of swing times in patients with CSVD (Ma et al., 2022). The effects of CSVD on the entropy of gait (Karmakar et al., 2007) were also analyzed. Permutation entropy analysis revealed a trend for a less ordered gait pattern in the CSVD group. However, neither *PE*_T_ nor *PE*_L_ were able to detect significant CSVD-related differences in the time series of the observed step parameters.

Our findings provide additional evidence that altered gait patterns can predict cognitive deficits in older patients. Increased gait variability was found to predict alterations in sustained attention in elderly patients with CSVD. Important in that regard is that attention is implicated in the control of locomotion in older adults. We posit that the presence of microbleeds and/or silent brain infarcts can affect both gait abnormalities and cognitive processes in a similar pattern, contributing to these associations. In light of the increased step length variability during dual task among CSVD patients with higher WMH burden (*p* = 0.008), these relationships apparently depend on the extent and spatial distribution of pathologies revealed by MRI. Results of the Mayo Clinic Study of Aging show that alterations in a number of gait parameters may predict age-related impairment in several domains of cognition (Savica et al., 2017). However, we did not find a significant correlation between the gait abnormalities studied and many other measures of cognitive performance in older patients with CSVD. It can be ascribed to the heterogeneous manifestations of CSVD in affected older individuals. Further studies on larger cohorts are evidently needed to characterize in more details the individual effects of CMHs, WMHs, and lacunar infarcts on different domains of cognition and on different gait parameters (Valkanova et al., 2018; Cai et al., 2022).

The current study has several limitations, including the small sample size, which did not allow for control for the effect of comorbidities or for a comprehensive assessment of the potential impact of CVSD localization. It was however only of concern for type 2 diabetes mellitus which may have biased the differences between the patient and control group due to its unbalanced distribution and impact on cerebrovascular and cognitive impairment (Liu et al., 2018; Teng et al., 2021). CSVD patients with diabetes (*n* = 5) had similar gait parameters and slightly worse RVPA performance (*p* = 0.048) compared to the rest of the CSVD group. However, the effect sizes of our main findings suggest an independent contribution of CSVD to the endpoints, and predicts more significant gait pattern differences related to CSVD and a stronger association between gait alterations and cognitive impairment in elderly patients with CSVD in larger samples. The lack of MRI for control participants should also be acknowledged as a limitation, albeit there were no cognitively impaired in this patient group and the presence of WMH reaching a Fazekas score greater or equal than two is less likely among community-dwelling older adults. The third limitation is related to the cognitive measurements used since we relied solely on computerized cognitive testing, which may not fully map onto well-established neuropsychological tests (Smith et al., 2013), and these tests were administered separately from the gait assessment. Fourth, gait recordings obtained from longer walking sessions would allow for estimating other well-established parameters of gait dynamics, including the scale-free alpha parameter obtained by detrended fluctuation analysis (Dutta et al., 2013) or alternative entropy measures (Karmakar et al., 2007). Finally, a more challenging dual task may have enabled a more sensitive analysis of the relationships between subtle alterations in gait parameters and cognitive performance.

## Conclusion

Taken together, older adults with CSVD exhibit significant, subclinical gait abnormalities that can be captured in a constellation of step time variability and step length asymmetry enhanced by a dual task paradigm. Our current data also suggest that older adults with CSVD have reduced attentional resources, which are exhausted during dual task. Finally, we found that higher gait variability associates with impaired cognitive performance in older adults with CSVD. We therefore propose that gait variability and symmetry parameters could be developed as biomarkers of CSVD-related cognitive dysfunction. Further research, addressing the limitations of this study, should be conducted in a larger population with extended follow-up in order to validate these findings as an early indicator of CSVD-related cognitive dysfunction.

## Data availability statement

The raw data supporting the conclusions of this article will be made available by the authors, without undue reservation.

## Ethics statement

The studies involving human participants were reviewed and approved by Institutional Review Board of the University of Oklahoma Health Sciences Center. The patients/participants provided their written informed consent to participate in this study.

## Author contributions

PM drafted the first version of the manuscript. PM, SD, and CBP evaluated gait assessments, PM, SD, JH, and AY evaluated cognitive assessments. CO, TC, AL, and AY performed all assessments that required patient contact. TC, AL, ST, PB, AN-T, AC, and ZU contributed to study design. ZU, AK, CIP, and AY acquired funding for the study. AK and CIP helped with patient enrollment and clinical data collection. ZU and AY supervised the research project. All authors read and critically reviewed the manuscript.
